# Evaluating the significance of left ventricular midwall fibrosis detected by late gadolinium enhancement imaging on left ventricular functional remodelling in dilated cardiomyopathy

**DOI:** 10.1186/1532-429X-18-S1-P283

**Published:** 2016-01-27

**Authors:** Upasana Tayal, Simon Newsome, Ricardo Wage, Aamir Ali, Brian Halliday, Zohreh Farzad, Dudley J Pennell, Stuart Cook, Sanjay Prasad

**Affiliations:** 1 grid.439338.6Royal Brompton Hospital, London, United Kingdom; 2 grid.7445.20000000121138111Imperial College, London, United Kingdom; 3 grid.8991.9000000040425469XDepartment of Medical Statistics, London School of Hygiene and Tropical Medicine, London, United Kingdom; 4 Duke University, Singapore, Singapore

## Background

Dilated cardiomyopathy (DCM) is associated with a 20% 5 year mortality rate.

We identified that the presence of left ventricular (LV) midwall fibrosis, detected by CMR late gadolinium enhancement (LGE), was associated with a 5 fold increased risk of cardiovascular death and adverse events, independently of established predictors of outcome.

The natural course of DCM is however variable; up to 15% of patients may recover function, with normalisation of left ventricular ejection fraction (LVEF).

We sought to evaluate whether LGE, a robust predictor of clinical outcome, was predictive of remodelling in DCM.

## Methods

130 prospectively recruited patients with DCM (mean age 53.9 years, 65% male) underwent baseline and follow up CMR studies using a 1.5T Siemens scanner.

The presence of midwall fibrosis was evaluated through the administration of 0.1 mmol/kg of gadolinium based contrast agent (Gadavist, Schering AG, Berlin). Images were obtained using an inversion-recovery prepared gradient echo sequence in standard long and short axis views, with inversion times optimised to null normal myocardium. Images were repeated in two separate phase encoding directions to exclude artefact.

ANCOVA was used to evaluate the significance of LGE on predicting the follow up LVEF, controlling for the baseline LVEF and other covariates. Students t-test was used to compare LVEF between groups with and without LGE. All statistical calculations were performed using R.

## Results

**Figure 1:**
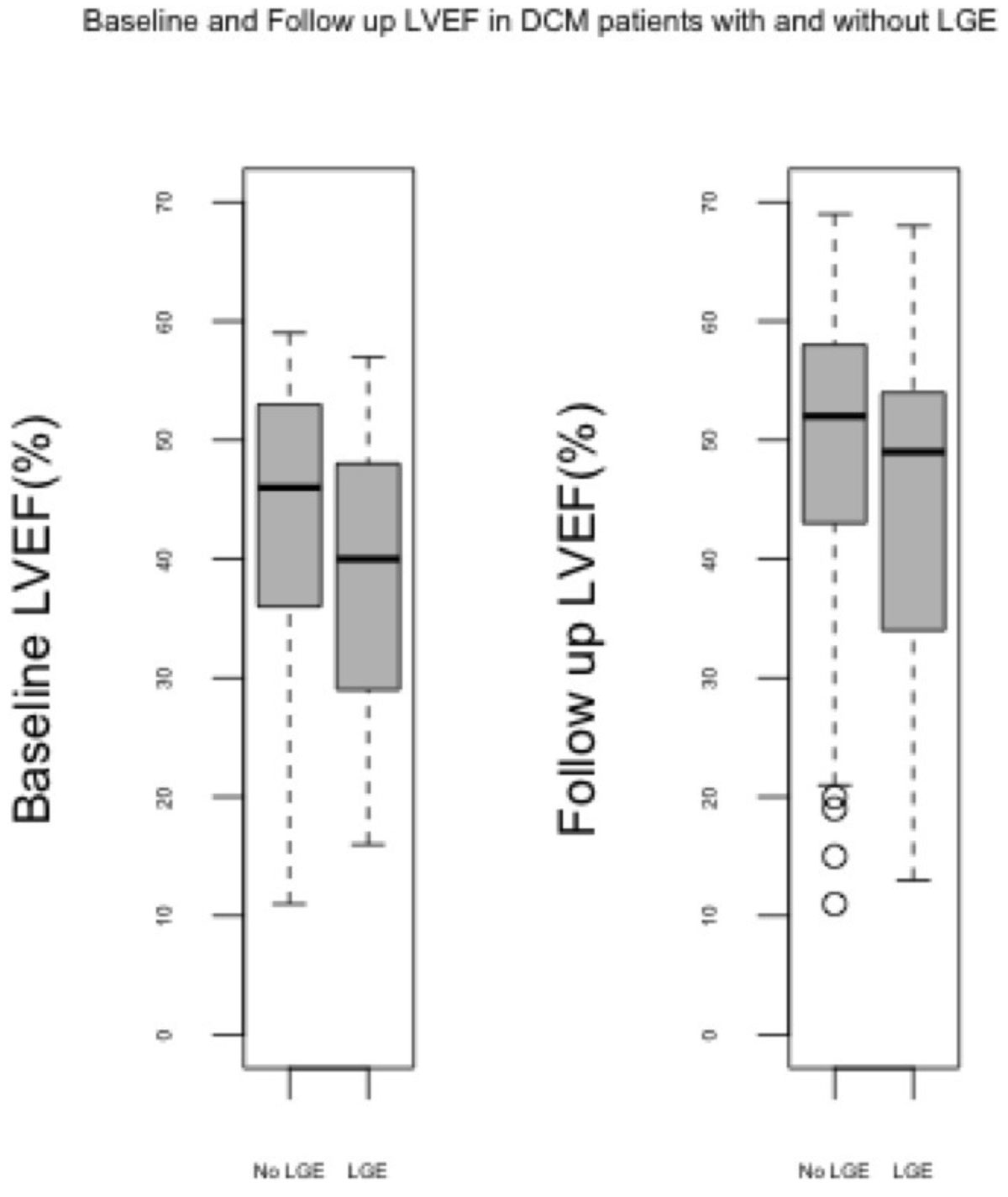
**Baseline and follow up left ventricular ejection fraction (LVEF) in DCM patients stratified by the baseline presence or absence of midwall fibrosis**. LGE- late gadolinium enhancement.

Median follow up interval was 2.7 years between scans (IQR 1.4-4.7 years). Fifty patients (38%) had evidence of LV midwall fibrosis at baseline. The mean baseline LVEF in patients with LGE was 39.3% and 43.6% in patients without LGE (p=0.05; Figure 1). The mean interval change in LVEF in patients with LGE was 5.1% and 4.8% in those without (p=0.9).

Controlling for baseline LVEF, initial analysis using ANCOVA found that those patients with DCM and LGE had a 1.6% lower follow up LVEF than those without, but this was not significant (p=0.429, 95% confidence interval -5.9% to 2.5%).

When the additional variables were also included in the model (age, gender, ethnicity, LV and RV end diastolic and end systolic indexed volumes, baseline RVEF, mitral regurgitation, history of AF and hypertension, baseline pulse rate and blood pressure, NYHA class, and medication use- diuretic, beta blocker, ACE inhibitor and aldosterone antagonist), the estimated effect of LGE was a 2% lower follow up LVEF, but this was not significant (p=0.444, 95% confidence interval -7.5% to 3.3%).

## Conclusions

Whilst LGE is a robust predictor of adverse outcome in DCM, in this cohort there was no evidence to suggest that the presence of LGE predicted left ventricular remodelling.

These findings highlight that predictors of outcome and remodelling in DCM are not interchangeable. Our future work will explore these findings in a larger cohort with a quantitative evaluation of midwall fibrosis and to evaluate the relationship between reverse remodelling and clinical outcome in DCM.

